# Implicit Theories of Emotional Intelligence and Students’ Emotional and Academic Outcomes

**DOI:** 10.1177/00332941231183327

**Published:** 2023-06-10

**Authors:** Ana Costa, Luísa Faria

**Affiliations:** 166419 Faculty of Psychology and Education Sciences, University of Porto/Portugal, Porto, Portugal

**Keywords:** Implicit theories of emotional intelligence, emotional intelligence, emotions towards school, academic achievement, secondary school

## Abstract

In this study, we addressed the relevance of implicit theories of emotional intelligence (ITEI) to students’ emotional and academic outcomes throughout secondary school. During a three-wave longitudinal survey (10^th^–12^th^ grades), 222 students, ages 14–18 years old at the first round of data collection (*M*_age_ = 15.4, *SD* = 0.63) and mostly female (58.6%), completed questionnaires on ITEI, emotional intelligence (EI; ability and trait), and emotions towards school. The results provided evidence for the relation of ITEI with EI (ability and trait) in the following year and their extended link with students’ emotions towards school and academic achievement (Portuguese academic grade) at the end of secondary school. In addition, ability and trait EI mediated the link of entity ITEI and negative emotions and achievement. The findings suggest the importance of fostering more dynamic ITEI among students as a mean for enhancing emotional and academic outcomes.

## Introduction

Students’ experiences in school are filled with strong academic, emotional, and social stimuli. The transition to secondary school in particular adds challenges and opportunities to adolescents’ developmental path: As they adapt to the increasing autonomy and changes and/or implementation of new roles in familiar and social settings (Lerner, & Steinberg, 2009), students’ bear a progressive responsibility for their educational trajectories and outcomes (Benner, 2011; Benner & Graham, 2009).

As is well recognised in the field of education, emotional and motivational factors affect students’ adaptation, well-being, relationships, accomplishments and, ultimately, their development (Gogol et al., 2017; Zins et al., 2007). Therefore, students’ experiences at academic and emotional levels will likely influence their forthcoming educational and professional trajectories.

Furthermore, because the literature has established that a person’s specific beliefs about different attributes can strengthen or undermine diverse outcomes (e.g., Dweck & Yeager, 2019; Karlen et al., 2019; Zhang et al., 2023), the construct of implicit theories (ITs) has gained increased visibility. In the academic context, a series of studies has addressed the relevance of students’ ITs of intelligence (Dweck, 2017; Dweck & Yeager, 2019; Gál et al., 2023; Karlen et al., 2019; Yeager et al., 2019), yet so far the ITs of emotion-related attributes have not been well supported. In addition, emotional intelligence (EI) has been systematically identified as a crucial determinant of students’ emotional and academic outcomes, and there is evidence that EI can be nurtured and developed (Mattingly & Kraiger, 2019). Within this framework, implicit theories of emotional intelligence (ITEIs) constitute an attribute worth investigating because of their potential relevance to enhancing or undermining students’ emotional self-perceptions and abilities, which affect their adaptation, well-being, and achievement. On the basis of evidence of the potential effects of ITs in the academic context, in the present study we addressed the less explored ITEIs and investigate whether this type of IT can relate to students’ emotional (EI and emotions towards school) and academic (Portuguese, biology, and mathematics grades) outcomes throughout secondary school.

### Implicit theories

People can hold specific beliefs about the nature of diverse individual and group attributes (e.g., intelligence, emotion, personality, relationships, and health). Those who hold beliefs about the nonmalleability or predeterminism of different domains are considered *entity* or *fixed theorists*, whereas those who understand such attributes to be malleable and, at some point, changeable are referred to as *incremental theorists* (Dweck et al., 1995; Dweck & Yeager, 2019). Naturally, the implicit mindset that is used to understand specific attributes maps and reinforces an individual’s particular understanding and their motivational and behavioural responses in specific situations (Crum et al., 2013; Dweck & Yeager, 2019). On the one hand, because entity theorists tend to perceive attributes as more stable and internal, whenever they face challenges they exhibit less motivation to regulate their behaviour; on the other hand, incremental theorists consider attributes to be more flexible and dependent on specific contexts and therefore are more likely to engage in assertive self-regulation strategies to adapt their behaviour (Dweck & Yeager, 2019).

Until now, the literature in the field of ITs has provided strong evidence that having incremental ITs about diverse attributes (e.g., leadership, personality, intelligence, or emotions) is associated with positive effects, for instance, increased well-being, mental health, social relationships, academic achievement, and job performance (Burnette et al., 2010; Costa & Faria, 2018; Dweck, 2017; Gál et al., 2023; Tamir et al., 2007; Yeager et al., 2019; Zhang et al., 2023), whether in youth or adulthood (Costa & Faria, 2018; Dweck, 2017).

Especially in the field of ITs of intelligence, an extensive amount of research has indicated that incremental theories of intelligence are consistently related to favourable outcomes: In Burnette and colleagues’ (2013) meta-analytic review the results suggested that incremental theories positively predicted students’ learning goals, mastery-oriented strategies, and positive expectations of success, whereas other studies have indicated students’ higher motivation and engagement (De Castella & Byrne, 2015) or have minimised the impact of academic adversities on students (Gál et al., 2023). The literature has also suggested that ITs can interfere with self-regulatory processes that predict achievement (Burnette et al., 2013) and task performance (Tabernero & Wood, 1999).

Thus, ITs are recognised as a relevant variable within the academic context, because they can influence and shape students’ self-perceptions and behaviours, which in turn will affect their attainment and performance. Research has also highlighted how higher levels of incremental ITs are related to students’ positive outcomes.

### Emotional intelligence in school

EI has achieved a relatively prominent position in the current state of the art based on increasing empirical evidence for its positive influence in diverse contexts. Considered a multidimensional phenomenon, EI encompasses a person’s perceptions, appraisals, and expressions of emotion; use of emotion to facilitate thought; and understanding and regulation of emotions in themselves and others (Mayer & Salovey, 1997). In the field, two main theoretical approaches have been established: (a) *ability EI*, which is considered to be a set of emotion-related cognitive skills and functions as a parallel to other types of intelligence and therefore is assessed by objective performance measures or tests (Mayer, Salovey, & Caruso, 2008), and (b) *trait EI*, which is related to the realm of personality and traits and is theorised to be a constellation of emotional perceptions typically assessed via self-report measures (Petrides et al., 2007).

Research has revealed that students’ emotional environment and experiences in school can influence their learning and progress, not only by undermining specific cognitive learning processes (e.g., attention, perception, memory, or decision making; Clore & Palmer, 2009; Loewenstein & Lerner, 2003) as a consequence of negative emotions but also by affecting their engagement and motivation as well as their learning strategies (Christopher & Allen, 2021; Linnenbrink, 2007). In fact, trait and ability EI have been associated with positive learning processes, such as motivation, engagement, adjusted academic goals, and academic achievement and performance (Costa & Faria, 2015; MacCann et al., 2020; Maguire et al., 2017; Sánchez-Álvarez et al., 2020; Trigueros et al., 2019), and with the provision of support for emotion-related intrapersonal and interpersonal dimensions, such as stress regulation, well-being, positive emotions towards school, less school refusal conduct, and positive school interactions (Brackett et al., 2011; Díaz-Herrero et al., 2018).

Thus, EI has been particularly valued in the field of education because the ability to control and manage emotions that arise in everyday schooling is considered a relevant competency that helps students face challenges and adapt throughout their learning and achievement trajectories (Goetz & Bieg, 2016).

### Implicit theories of emotional intelligence

One of the domains more recently explored is that of ITs related to emotion-related attributes, mainly because of the relevant effect emotion has on individuals’ psychological and social functioning (King & dela Rosa, 2019; Tamir et al., 2007). In the field of ITs of emotion or emotion-related attributes there is empirical evidence that individuals who hold an incremental IT, mostly because of their perspective on dispositional and transitional emotional states, are more likely to adopt better emotion self-regulation strategies and have higher experiences of positive emotions and well-being compared with their entity theorist counterparts (Ford & Gross, 2018; King & dela Rosa, 2019; Shih, 2011; Zhang et al., 2023).

In this line, in the present study we intended to contribute to the field by exploring the effects of ITEIs, which refer to people’s beliefs about their ability to control or change their emotion skills and perceptions, namely, their emotional expression and perception, and their understanding and use of emotions to facilitate thought and emotional regulation, in the secondary school context. This type of IT differs from those of emotion mainly because it focuses not on the possibility of an individual changing their emotions and emotional states over time but on the possibility of changing their emotion set of skills, whether the way they express, perceive, use, or manage emotions, through effort and investment or over time. It also differs from trait EI in that the latter refers to the individual’s perception of emotional competence rather than their ability to change that competence over time.

The literature in this topic is scarce. Cabello and Fernández-Berrocal (2015) showed that individuals who hold incremental theories of EI had better EI performance, which could be explained by the fact that this type of theorist believes that EI can be changeable and, when facing new or difficult emotional experiences, were perhaps more likely to perceive such experiences as a necessary means of developing and acquiring new abilities. In another study with Portuguese secondary students, and the first in this context that compared the predictive association of ITEIs on both trait and ability EI, Costa and Faria (2020b) found a positive relation of ITEIs and students’ trait EI but not with their ability EI. Another interesting finding is that, although ITEIs did not exhibit a direct link to students’ achievement (grade point average), they positively affected all the emotional dimensions of trait EI, which in turn predicted academic achievement. These results indicate that ITEIs can play a role in students’ academic achievement by reinforcing their perceptions of emotional competence, which in turn might foster their academic progress (Costa & Faria, 2020b).

Tamir and colleagues’ (2007) study of college students in the field of ITs of emotion is particularly relevant in informing about the domain specificity of ITs, highlighting that ITs of emotion were associated with emotional and social adjustment. Their results also revealed that ITs can have an effect on individual’s self-regulatory processes. Indeed, at the beginning of college, incremental theorists reported higher self-efficacy in emotion regulation (*r* = .24, *p* < .05) and adopted more cognitive reappraisal strategies (*r* = .35, *p* < .05). On the other hand, at the end of the first year of college, the previously identified incremental theorists students described higher levels of well-being (*r* = .24, *p* < .05) and social adjustment (*r* = .12, *p* < .05) and fewer depressive symptoms (*r* = −.15, *p* < .05) and loneliness (*r* = .16, *p* < .05). In this study, self-efficacy in emotion regulation was found to mediate the link between ITs of emotion and emotional outcomes; that is, when individuals hold a general belief that emotions are flexible and changeable they tend to believe that they can actually change their own emotions, initiating a self-regulatory process.

Thus, because of the potential adaptive role of emotion and emotion-related attributes in regard to students’ educational paths, researchers in the field have made efforts to deepen the relevance of ITs of emotion and of EI in the academic setting. However, although some studies have supported the association of incremental perspectives and positive outcomes, research in the field is still scarce and mainly based on cross-sectional designs. Within this framework, in this study we explored the effects of students’ ITEIs on different outcomes throughout the complete secondary school cycle.

In the field of ITs, different studies have explored whether such theories present differences according to gender group. In the domain of ITs of intelligence, recent meta-analytic reviews have not established differences between gender groups, or they have found inconclusive results (Burnette et al., 2013; Costa & Faria, 2018). In the realm of emotion-related attributes, the number of studies that have explored gender as a moderator is still too small to draw conclusions: In one study, women were found to have a higher likelihood of being incremental theorists of emotion and of EI (Cabello & Fernández-Berrocal, 2015); however, in another study gender was not related to ITs of emotion (Romero et al., 2014), and a different study found no evidence of gender-based differences of ITEIs on students’ emotional outcomes in a Portuguese context (Costa & Faria, 2020b). Therefore, research in the field should continue to address potential gender-based differences.

### The current study

On the basis of the evidence that ITs can play a role over time that stands out during particularly challenging transitions, in this study we explored their relevance throughout the 3-year Portuguese secondary school cycle.^1^ This study extends the literature on the recently new ITEI field, providing empirical evidence of their implications for students’ future emotional and academic outcomes and for addressing secondary school as a particular challenging academic stage.

Because ITs relate to both perceptions and performance, in this study we explored whether ITEIs can be a well-established predictor of students’ adaptation and achievement, in this case, students’ EI. To fully capture and distinguish the potential effect of ITs on students’ EI perceptions and performance, we included the trait and ability conceptualisations of EI and different measurements. We also explored whether ITEIs have a lasting direct and/or indirect relation to relevant indicators of students’ academic achievement at the end of the secondary school, such as emotions towards school and academic achievement.

Because other studies have found a potential mediating effect of self-regulatory processes on the link between ITs (of intelligence, Burnette et al., 2013; of emotion related, Tamir et al., 2007) and academic and emotional outcomes, we explored potential mediators between ITs of EI and emotional and academic outcomes.

The goals of the study were to explore the following:

a) Whether and how ITEIs predict students’ EI. We predicted that students’ incremental ITEIs would predict positively, and the entity perspective negatively, their EI self-perceptions (trait EI) and objective performance (ability EI) for the following year (Hypothesis 1 [H1]).
b) Whether ITs predict students’ academic and emotional outcomes at the end of secondary school cycle. We predicted that incremental ITEIs would predict positively, whereas the entity perspective would predict negatively, students’ academic achievement in different subjects and emotions towards school at the end of secondary school (Hypothesis 2a [H2a]); we also predicted that ITEIs would be more related to students’ emotions towards school than students’ academic grades, based on ITs’ domain specificity (Hypothesis 2b [H2b]).
c) Whether EI is related to students’ emotional and academic outcomes in the last year of secondary school. We predicted that both trait and ability EI are positively related to positive emotions and negatively to negative emotions towards school and positively associated with students’ academic achievement in the following year (Hypothesis 3 [H3]).
d) Whether EI mediates the link between ITs and academic and emotional outcomes. We predicted that both trait and ability EI would mediate the relationship of incremental (positively) and entity (negatively) ITEIs and students’ emotions towards school and academic achievement (Hypothesis 4 [H4]).
e) Whether students’ gender acts as a moderator of the association between ITs and their emotional outcomes and academic achievement. We predicted that girls and boys would not differ in regard to the relationship between incremental and entity ITs and their emotions towards schools and academic achievement (Hypothesis 5 [H5]).

## Method

### Participants

A total of 222 Portuguese secondary school students (41.4% male) were surveyed across three time points (42.4% of the initial student sample in 10^th^ grade [*n* = 523] and 64.8% of the student sample in 11^th^ grade [*n* = 343]), 1 year apart each, by using a longitudinal design. In the first phase of data collection, in 10^th^ grade, the participants were between ages 14 and 18 years (*M*_age_ = 15.4, *SD* = 0 .63). They constituted a convenience sample, from a total of seven public high schools in a large urban city in the north of Portugal. Students were recruited within different classrooms in each school, previously selected by the schools’ administration. They were enrolled in different academic courses (76.1% in science and technology, 19.4% in languages and humanities, and 4.6% in other courses), and the majority of the sample was of a high parental socioprofessional status^2^ (37.7% high, 28.8% middle, and 33.5% low status).

### Measures

#### Implicit Theories of Emotional Intelligence Scale

Based on Dweck’s (1999) original theorisation of ITs of intelligence, this scale, which evaluates individuals’ ITs about the malleability of EI, was developed and adapted by (Costa & Faria, 2020a) for the Portuguese secondary school context. The ITEIS includes a total of 12 items rated on a 6-point Likert-type scale, ranging from 1 (*strongly agree*) to 6 (*strongly disagree*), with six incremental IT items (e.g., ‘Every time I learn with new experiences, my emotional intelligence increases’) and six entity IT items (e.g., ‘My emotional intelligence is something about me that I personally can’t change very much’). The ITEIS has a two-factor structure; one factor represents the entity theory and the other represents the incremental theory (factorial correlation of −.67). In this study, the entity and incremental factors were treated as separate variables. The scale exhibited good fit indices (comparative fit index [CFI] = .97, Tucker–Lewis Index [TLI] = .96, root-mean-square error of approximation [RMSEA] = .061, standardised root-mean-square residual [SRMR] = .035; Costa & Faria, 2020a, 2020b). In this study, the ITEIS presented very good reliability (total scale α = .87, entity scale α = .86, incremental α = .80). The total scale and scale dimensions values were generated through summing items.

#### Emotional intelligence measures

##### Emotional Skills and Competence Questionnaire

The Emotional Skills and Competence Questionnaire (ESCQ; Takšić et al., 2009) is a 42-item self-report measure based on the trait EI model that captures the perceptions of individuals across three dimensions: Perceive and Understand Emotion (14 items, e.g., ‘When I see how someone feels, I usually know what has happened to him or her’), Express and Label Emotion (14 items, e.g., ‘I am able to express my emotions well’), and Manage and Regulate Emotion (14 items, e.g., ‘When I am in a good mood, every problem seems solvable’). Based on Mayer and Salovey’s EI model (1997), this measure was developed by Takšić et al. (2009) for the Croatian context and adapted to the Portuguese context by (Faria et al., 2006). Adapted and translated for diverse cultural contexts, the ESCQ has shown adequate psychometric proprieties in terms of underlying structure, positive correlations between the dimensions (between .49 and .54), good reliability (between .72 and .91; Faria et al., 2006; Takšić et al., 2009), cross-cultural measurement invariance (Croatian original scale and Portuguese adapted version; Costa et al., 2016) and good fit indices (normed fit index [NFI] = .93, CFI = .94, root-mean-square residual [RMR] = .04, RMSEA = .04; Stocker & Faria, 2012). In this study, the ESCQ presented very good reliability (total scale = .90; dimensions range from .72 [Manage and Regulate Emotion] to .89 [Express and Label Emotion]). The total scale and scale dimensions values were generated through summing items.

##### Vocabulary of Emotions Test

The Vocabulary of Emotions Test (VET; Takšić et al., 2003) is a 35-item EI measure based on the third dimension of the ability EI model: Understand Emotion. As a performance measure, this test evaluates emotional knowledge using a vocabulary test format. Developed by Takšić et al. (2003), this test has the same format as any other classic vocabulary test, yet the items refer to emotionally saturated target words. The task requests that the respondent choose an adjective (from six options presented, e.g., ‘sad’, ‘lonely’, ‘angry’, ‘merry’, ‘satisfied’, or ‘nothing listed’) that has the closest meaning to the target word (emotion, e.g., ‘happy’). Correct answers for the test are based on dictionary definitions. The original version of the VET exhibited good psychometric properties: moderate correlations with other intelligence tests (California Tests of Mental Maturity Vocabulary Test, *r* = .67, *p* = .00, and Logical Thinking, *r* = .33, *p* = .00), and with EI tests (Analysis of Emotions Test, *r* = .46, *p* = .00), and explains 44% of the specific variance over and above classic intelligence tests. This measure was adapted to the Portuguese context and has adequate psychometric proprieties: item difficulty (*M* = .55; *SD* = .22), reliability above .71, and differential validity for gender and the cultural context (Costa et al., 2011). In this study, the VET exhibited very good test–retest reliability (*r* > .63). The total scale value was generated through summing items (correct answers).

#### Students’ emotions towards school

Based on the Academic Emotions Questionnaire (Pekrun et al., 2011), this self-report measure assesses students’ emotions towards school and comprises two dimensions: positive emotions towards school (four items, e.g., ‘I feel happy’, ‘I feel proud’) and negative emotions towards school (five items, e.g., ‘I feel bored’, ‘I feel ashamed’), answered with a 6-point frequency response scale (range: 1 = *never* to 6 = *always*). This scale has previously demonstrated satisfactory psychometric proprieties (Costa & Faria, 2020a) and in this study exhibited acceptable internal consistency (α for the total scale = .69). The total scale and scale dimensions values were generated through summing items.

#### Students’ Academic Achievement

Students’ academic marks were obtained from school records for the last academic period of each year for the 3 consecutive years of secondary school (10^th^, 11^th^, and 12^th^ grades). From the available subjects (a total of six mandatory academic subjects in secondary school), and to represent different scholastic dimensions, students’ verbal/language (Portuguese), mathematics, and science (biology) final academic achievement grades were collected. In the secondary school cycle, academic achievement grades range from 0 to 20.

### Procedure

All participants individually filled out the paper-and-pen questionnaires in their classrooms during rounds of collective administration after receiving brief group instruction on the answer formats. The survey administration occurred typically from February to March (second period of secondary school) of each academic year. The students were informed about the voluntary nature of their participation and the confidential nature of the study. They were also informed that nonparticipation did not entail any type of consequence. Underage students had to provide informed parental consent to participate in the study, and students who were of age signed their authorisation to participate. Before administering the instruments, we explained to the participants the meaning of the terms *intelligence* and *EI* to make sure all of them understood the scales’ items. They took, on average, 25 min to complete the questionnaires. The administration-counterbalance technique was not applied in this study. This study received a favourable opinion from the Portuguese National Data Protection Commission, Directorate-General for Education, and Faculty’s Ethics Committee.

### Data analyses

**Figure 1.**
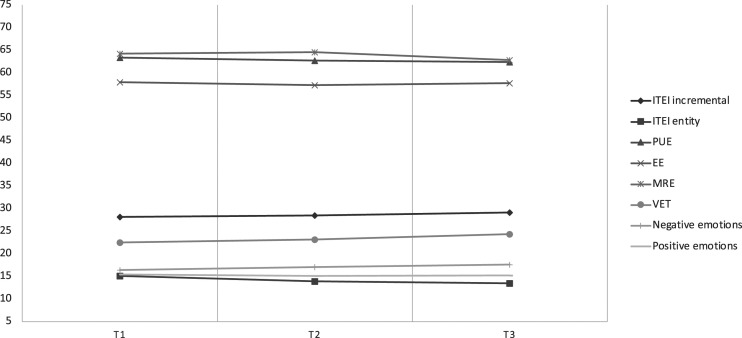
Trendline for mean values of variables in analysis across the three time-points. Note. ITEI-Implicit Theories of Emotional Intelligence; PUE-Perceive and Understand Emotion; EE- Express Emotion; MRE-Manage and Regulate Emotion; VET-Vocabulary of Emotions Test; Negative Emotions -Negative Emotions towards School; Positive Emotions -Positive Emotions towards School.

All statistical procedures were conducted using AMOS Version 26.0. We used path analysis to explore whether students’ incremental and entity ITEIs in the first year of secondary school (10^th^ grade; Time 1 [T1]) had a direct link to their EI self-perceptions and objective performance in the next academic year (11^th^ grade; Time 2 [T2]) and whether such ITs predicted students’ emotions towards school and academic achievement in the last year of secondary school (12^th^ grade; Time 3 [T3]; see Figure 1). Students’ entity and incremental beliefs were treated as separate variables and included in the model (T1) to test unique effects and to provide detailed information on the implications and comparative predictive power of each type of belief. The path analysis was conducted using maximum-likelihood estimation, which is more robust to deviations from normality. The model’s goodness-of-fit indices considered were nonsignificant χ^2^ statistics, CFI, goodness-of-fit index (GFI), TLI (best if above .95), and RMSEA (best if .08 or less with a 90% confidence interval [CI]; Hu & Bentler, 1999).

To account for missing data, we used stochastic regression imputation (Little & Rubin, 2002) that imputes values for each case by drawing at random from the conditional distribution of the missing values given the observed values, with the unknown model parameters set equal to their maximum-likelihood estimates.

To conduct the mediation analyses, we first separated the direct links of the independent variables (ITEIs) to the dependent variables (academic achievement and emotions towards school), of the independent variables to the mediator variables (trait and ability EI), and of the mediator variables to the dependent variables, each of which should be significant in order to pursue the mediation procedure. Next, to test for indirect effects, we conducted a bootstrapping analysis (*N* = 5000 samples). When the bootstrapped 95% CI for the point estimate does not include 0, the effect is considered significant (Preacher & Hayes, 2008).

We conducted a multigroup moderation analysis to test for a potential moderating effect of gender in the model. In cases where χ^2^ differences in the model comparisons (model-free structural paths vs. structural paths constrained to be equal across groups), the model differs across the tested groups.

## Results

### Descriptive analyses

Descriptive statistics (means, standard deviations) and the correlation matrix for the variables in this study are displayed in Table 1. In general, the ITEI dimensions had significant correlations with trait EI and students’ emotions towards school. In particular, ITEIs were modestly associated with higher levels of trait EI, and entity ITEIs were modestly associated with lower levels of trait EI. Having positive emotions towards school was moderately correlated with higher levels of incremental ITEIs and negatively associated with higher levels of entity ITEIs. Negative emotions towards school were moderately correlated with higher levels of entity theories. Modest to moderate negative associations were found for entity ITEIs and higher levels of the EI (trait and ability) variables and the Portuguese grade variable. In general, students’ academic achievement was associated only with higher levels of the ability EI measure, achieving moderate associations with higher Portuguese and math grades and modest associations with higher positive emotions towards school.

**Table 1.** Descriptive statistics and correlation coefficients for the studied variables.

Variables	*M(SD)*	1	2	3	4	5	6	7	8	9	10	11
1. Incremental ITEI	28.19 *(4.42)*	1										
2. Entity ITEI	15.08 *(5.67)*	−.64**	1									
3. PUE (trait EI)	62.72 *(8.40)*	.19**	−.14**	1								
4. EE (trait EI)	57.31 *(10.89)*	.13**	−.17**	.20**	1							
5. MRE (trait EI)	64.59 *(8.29)*	.18**	−.16*	.37**	.46**	1						
6. VET (ability EI)	23.10 *(4.11)*	.07	−.13*	.02	.03	.07	1					
7. PE	15.22 *(3.38)*	.18**	−.25**	.08	.12	.29**	.09	1				
8. NE	17.62 *(3.85)*	−.13	.33**	−.10	−.30**	−.32**	−.14*	−.44**	1			
9. Port	14.48 *(2.66)*	.10	−.20**	.09	.01	.11	.40**	.21**	−.10	1		
10. Math	13.89 *(3.39)*	.06	−.10	.04	−.05	.07	.34**	.19**	−.10	.85**	1	
11. Bio	17.53 *(1.83)*	−.02	−.05	.04	−.03	.03	.23*	.16*	−.07	.85**	.81**	1

*Note.* ITEI-Implicit Theories of Emotional Intelligence; EE- Express Emotion; PUE-Perceive and Understand Emotion; MRE-Manage and Regulate Emotion; VET-Vocabulary of Emotions Test; PE-Positive Emotions towards School; NE-Negative Emotions towards School; Port - Portuguese 12^th^ grade; Math - Mathematics 12^th^ grade; Bio- Biology 12^th^ grade. *N* = 222. **p < .001; *p < .05.

To explore the trajectory of each relevant variable in the analysis, we calculated the mean value across the three time points (see Figure1).

### Path analysis

**Figure 2.**
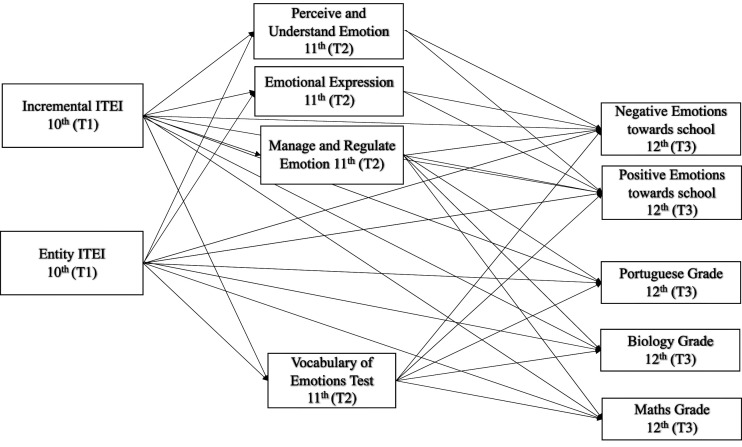
Path model depicting the prediction of students’ ITEI in 10^th^ grade (T1) on EI (trait and ability) in 11^th^ grade (T2) and on emotions towards school and academic achievement in 12^th^ grade (T3).

The proposed path model (see Figure 2) was tested and revealed unsatisfactory fit indices, χ^2^ (10, *N* = 222) = 97.871, *p =* .000; CFI = .887, NFI = .883, GFI = .935, RMSEA = .199.

Therefore, we conducted a subsequent model excluding the nonsignificant paths identified in the first model. The excluded nonsignificant paths were between students’ incremental ITEIs (T1) and emotional expression, VET score (in 11^th^ grade, T2) and positive emotions towards school and achievement in all subjects (in 12^th^ grade, T3), students’ entity ITEIs (T1) and perceptions and understanding of emotion and managing and regulating emotion (11^th^ grade, T2), and biology and math achievement (in 12^th^ grade, T3); trait EI (T2) and achievement in any subject (T3), and VET score (T2) and emotions towards school (positive and negative emotions, T3). The subsequent model exhibited an improvement in the model fit indices and provided very good model adjustment, χ^2^ (34, *N* = 222) = 35.661, *p* = .320; CFI = .998, NFI = .957, GFI = .972, RMSEA = .015.

**Figure 3.**
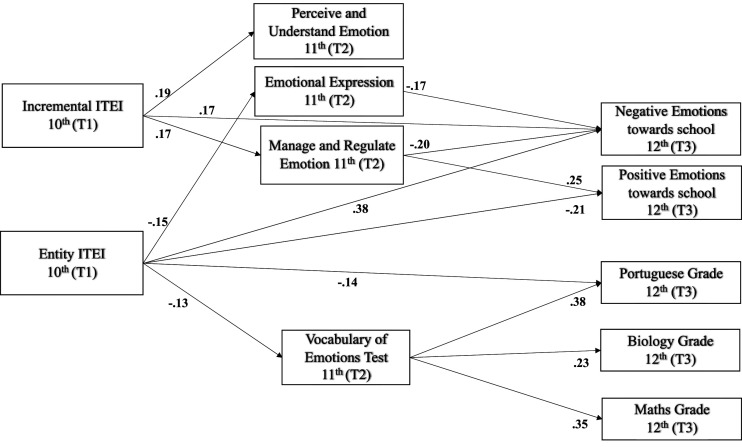
Path model depicting the prediction results of students’ ITEI in 10^th^ grade (T1) on EI (trait and ability) in 11^th^ grade (T2) and on emotions towards school and academic achievement in 12^th^ grade (T3). Note: ITEI-implicit theories of emotional intelligence; 10^th^-10^th^ grade; 11^th^-11^th^ grade; 12^th^-12^th^ grade. Path coefficients represented are standardized and significant (*p* < .05).

The results indicated that ITEIs in 10th grade (T1) positively predicted students’ perceptions of competence in perceiving and understanding emotion and managing and regulating emotion (trait EI, T2; see Figure 3), whereas entity ITEIs (T1) negatively predicted students’ perceptions of emotional expression (trait EI) and EI performance (VET) in the following year (T2).

The EI variables in 11^th^ grade also exhibited a link to students’ outcomes in the last year of secondary school. In particular, although students’ perceptions of emotional expression (T2) negatively predicted their negative emotions towards school in the following year (T3), their perceptions of managing and regulating emotions (T2) negatively influenced their negative emotions and fostered positive emotions at the end of secondary school (T3). On the other hand, the ability EI variable (VET) positively predicted students’ Portuguese language, math, and biology grades in 12^th^ grade (T3).

In addition, when we observed the effects over time in the model, we noted that incremental ITEIs (T1) exhibited an unexpected positive effect on students’ negative emotions towards school in the last year of secondary school (T3). The students’ entity ITEIs (T1) exhibited a generally negative impact on their outcomes in the last year of secondary school, including both positive emotions towards school and achievement in Portuguese (T3).

### Mediation analysis

The results of the mediation analyses are shown in Table 2. In general, the EI dimensions mediated the effect of entity ITEIs on students’ outcomes, although at a low level. In particular, it seems that EI attenuated entity conceptions of EI because EI weakened the negative link of entity ITEIs and Portuguese grades (β = −.05) and negative emotions towards school (β *=* .03, through an indirect effect). Moreover, managing and regulating emotion (trait EI) mediated the link between incremental ITEIs and negative emotions towards school (β = −.03).

**Table 2.** Total, direct and indirect effects of incremental and entity implicit theories of intelligence on students’ academic achievement and emotions towards school as mediated by trait and ability EI.

Predictor	Mediator	Outcome	Total Effect		Direct Effect		Indirect Effect	
			*b*	*β*	*b (SE)*	*β (SE)*	*b [95% CI Lower, Upper]*	*β [95% CI Lower, Upper]*
Entity ITEI	VET	Port	−.09	−.19	−.07 (.02)	−.14 (.03)	−.02 [−.05, −.01]	−.05 [−.11, −.01]
Entity ITEI	EE	NE	.27	.40	.26 (.05)	.38 (.07)	.02 [.00, .04]	.03 [.00, .07]
Incremental ITEI	MRE	NE	.12	.14	.15 (.06)	.17 (.07)	−.03 [−.10, −.00]	−.03 [−.12, −.00]

*Note. b*-non-standardized coefficients; *β* - standardized coefficients; SE-standard errors; 95% CI - 95% confidence intervals. Indirect effects are significant at the .05 level if the confidence intervals do not include zero; ITEI-implicit theories of emotional intelligence; VET-vocabulary of emotions test; EE – emotional expression; MRE – manage and regulate emotion; Port – Portuguese grade; NE – Negative emotions towards school.

### Multigroup gender moderation analysis

To explore whether the proposed model differed by student gender, we conducted multigroup moderation analyses. First, the model previously defined (see Figure 3) with the nonsignificant paths excluded was estimated for gender groups and provided good adjustment to the data, χ^2^ (64, *N* = 222) = 66.870, *p* = .379; CFI = .996, NFI = .924, RMSEA = .014. Next, to inspect for differences between gender groups, we constrained the model to be equal across gender groups. Model comparison exhibited a nonsignificant χ^2^ difference between the unconstrained and constrained models, which suggests that the final model did not differ between groups, in this case, gender, Δχ^2^(15, *N* = 130) = 10.308, *p* = .80.

## Discussion

In the present study, we sought to extend the evidence for the influence of ITEIs on secondary school, a particularly important cycle for determining students’ academic, professional, and personal goals. Indeed, this study is, to the best of our knowledge, the first to address this less explored type of ITs and to derive their implications for both academic and emotional outcomes throughout a complete academic cycle. The findings indicate that ITEIs have an effect on secondary school students’ EI, emotions towards school, and academic achievement. In particular, students’ entity beliefs of EI at the beginning of Portuguese secondary school predicted negatively their ability EI and trait emotional expression in the following year and had an extended negative impact on positive emotions towards school and Portuguese language grade at the end of secondary school. On the other hand, students’ incremental ITs in 10^th^ grade predicted positively their trait EI levels in the following year (11^th^ grade) and affected negatively the negative emotions towards school at the end of the cycle (12^th^ grade).

Our results support the domain-specific implications of ITs because the outcomes best predicted by ITEIs were related to students’ emotional experiences throughout secondary school.

### Implicit theories of emotional intelligence and emotional intelligence

Incremental ITEIs were positively related to secondary school students’ perceptions of competence in perceiving and understanding emotions and managing and regulating emotions in the following year, consistent with H1. These findings substantiate previous results in the literature that have shown that holding a more flexible perspective about the possibility of changing certain emotion-related attributes is associated with positive outcomes (Cabello & Fernández-Berrocal, 2015; Ford & Gross, 2018; King & dela Rosa, 2019; Romero et al., 2014; Tamir et al., 2007). In this study, the results, although at a low magnitude, indicated that believing EI to be an attribute that can be improved over time allows people to consider challenges and difficulties as presenting relevant emotional information about themselves and others that can promote and assist their emotional development. Moreover, this type of beliefs might have an extended effect on people’s actual emotional competencies over time.

As we expected on the basis of the literature, the entity perspective exhibited a negative predictive association with students’ perceptions and performances; that is, students who believed that EI could not be changed were more likely to consider themselves less able to express themselves emotionally and to expect themselves to perform worse in their understanding emotional content or situations. Moreover, only entity ITEIs were related to the performance EI measure, although the link was negative. These findings could perhaps be explained by the fact that the entity theorists tend to allocate higher importance to results and performance as the learning outcome, eliciting efforts that are focused on the exhibition of good performance and on the avoidance of bad performance (Dweck, 2012).

### Implicit theories of emotional intelligence and students’ academic and emotional outcomes

We found that ITEIs can have an extended role over time and relate to students’ academic and emotional outcomes at the end of the secondary school cycle, thus supporting H2. This result was expected on the basis of previous results (Cabello & Fernández-Berrocal, 2005; Tamir et al., 2007) because students’ beliefs about the possibility of controlling and regulating their EI could perhaps shape their actual emotional experiences over time. In particular, the entity dimension was the most relevant ITEI predictor, specifically in terms of the quantity and magnitude of predictions. Entity ITEIs predicted students’ emotions towards school at the end of secondary school and was the only dimension that predicted the objective performance indicators, such as students’ academic achievement (students’ Portuguese grade), therefore partially supporting H2a. As previously mentioned, the emphasis placed on the demonstration of results and products by the entity perspective might explain this finding, and perhaps enhance it by the end of secondary school, when students’ grades are particularly determinant of their subsequent academic and professional path.

The incremental ITs predicted positively students’ emotional perceptions of competence in the following year. In particular, this finding provides evidence that believing that emotional competencies are flexible and can be developed over time perhaps led students to be more available to engage in new emotional experiences and learn with them as well as to persevere and cope with emotionally challenging phases. Nonetheless, the fact that the incremental ITs did not affect students’ objective achievement (e.g., grades), as the entity theories did, was unexpected. The fact that, in this study, the entity beliefs had better predictive power could have shaded a potentially smaller effect of the incremental beliefs on achievement. However, the fact that both ITs influenced the emotional outcomes, in particular at the end of secondary school, were more strongly associated with students’ emotional experience towards school than was academic achievement gives support to the domain-specific implications of ITs (Costa & Faria, 2023; Tamir et al., 2007) previously hypothesised (H2b).

In addition, the results indicate that incremental ITEIs in turn exhibited a positive relationship with negative emotions towards school. This positive link of incremental ITEIs with students’ negative outcomes was unexpected but could reinforce the previous argument: that incremental theorists tend to experience more positive outcomes throughout secondary school compared with their entity theorist counterparts, but because incremental students focus on and value the process of learning more than performance it might be the case that, in the last year of this competitive cycle, they feel less well adjusted or adapted to what the educational system expects, which could result in the emerging of negative emotions.

### Emotional intelligence and students’ emotional and academic outcomes

Consistent with H3, EI was associated with students’ academic achievement and positive emotions towards school in the following year. This result is consistent with the existing literature that supports the positive associations between EI (trait and ability) and student endeavours and achievements (MacCann et al., 2020; Maguire et al., 2017; Sánchez-Álvarez et al., 2020; Trigueros et al., 2019). In particular, students’ emotional expression negatively predicted the potential negative emotions that could arise during the following year. It seems that the more students expressed their emotions and feelings in an adaptive way, the less likely they were to have negative emotions towards school in the next year. Furthermore, how students perceived their ability to manage and regulate emotion had a doubly significant effect: On the one hand, it negatively predicted their negative emotions, and on the other hand it positively reinforced their positive emotions towards school in the next year. These results indicate the detailed specificity of this EI dimension and its relevance to the regulation of emotional experiences and challenges during secondary school that protect students’ emotional experiences in the last and most important year of the cycle.

Ability EI — specifically, the ability to understand emotion — correlated with all academic subjects. In particular, ability EI was the only EI variable to demonstrate a predictive association with achievement, most likely because of the stronger association of the ability measure with the objective academic achievement indicators. In fact, the format of the test and the task requested were similar to paper-and-pen academic assignments (O’Connor & Little, 2003). Moreover, the higher association with the Portuguese language grade, found previously in another study that used this measure (Costa & Faria, 2015), could be caused by the specific test presented, which is like a classic vocabulary test and, to some extent, requires cognitive and academic resources similar to those of the tasks and evaluations in verbal or linguistic subjects.

### Emotional intelligence mediation

Another interesting finding is that the EI dimensions exhibited a particular mediating effect between ITEIs and students’ outcomes at the end of secondary school, supporting H4. Although self-efficacy in emotional regulation has mediated the link between ITs and emotional outcomes in other studies (Tamir et al., 2007), in this study, we noted that both ability and trait emotional self-efficacy acted as mediators of ITs and emotional and academic outcomes. Indeed, in this study the EI dimensions minimised the negative effect of an entity perspective of EI. On the one hand, the findings indicate that students who tend to view their EI as a relatively fixed attribute might be able to minimise those effects, using the developed skills of understanding emotion and emotional expression to attain improved emotional experience. In particular, the understanding emotion skill might facilitate students’ performance in the Portuguese language (mother tongue), for instance, when interpreting specific literary contents. Moreover, emotional expression might help cope with negative emotions towards school, reducing their manifestation. On the other hand, emotion regulation can turn the positive direct effect of incremental ITEIs on negative emotions towards school into a negative one. Students can perhaps manage their emotional state to cope with negative experiences they have had, thus limiting the expression of negative emotions towards school in the future.

In this study, EI mediated the negative consequences of students’ beliefs, although we should note that the mediating effects were small and call for further exploration. Another aspect worth highlighting is that ITEIs established a direct relation with students’ native language academic achievement, as previously observed, but their influence was more markedly indirect, through the promotion of EI abilities in the case of the ability to understand emotion.

### Gender moderation

No differences between gender groups were found in the predictive association between ITEIs and students’ academic and emotional outcomes throughout secondary school (H5). The fact that the secondary school challenges all students with new and diverse emotional, social, and academic experiences could support the idea that both gender groups have similar patterns of relations between the ITEIs and emotional outcomes. It also might be the case that in late adolescence students have stabilised their beliefs about emotion-related aspects and that these can be similar for boys and girls.

### Limitations

The present study has limitations that should be acknowledged and addressed in further research. First, the sample was relatively small, and a convenience sample, which limits the generalisability of the results and reinforces the need for replication studies. In addition to the analysed variables, other relevant academic or social indicators, such as students’ attendance rates, behavioural conduct, interpersonal relationships, or social support, could have been included to extend the predictive power of ITEIs in this context. On the other hand, the predictive association between ITEIs and student outcomes might have been decreased had other salient student variables been included. Second, traditional transversal academic subjects were selected as proxies for academic achievement, but different types of subjects related to other vocational interests, such as arts, economics, and sports, could have been integrated and explored. This would have been the case in the present study if the sample had been equitably distributed across subjects.

Moreover, we assessed the students’ ability EI with a test that focused on the dimension of understanding emotion. Although a more fine-grained level of analysis could produce more detailed results, ideally further research will include other dimensions, such as emotional regulation, in the assessment of ability EI. On the other hand, the trait EI measure was not specific to the academic context, which could have lessened the effect of EI on academic outcomes or masked possible mediation effects. It should also be mentioned that the reliability of the Students’ Emotions Towards School scale was below what was expected; thus, elaborations based on these results should be made with caution. Another aspect worth exploring in further research is other possible mediating factors that link ITEIs to emotional and academic outcomes. Future research should also provide insight into the link between ITEIs and students’ outcomes in earlier academic stages, when there is a broad multiplicity and diversity of students across emotional, motivational, and scholastic levels.

### Implications for the educational context

Our findings have practical implications for educational contexts. There is growing evidence for the need to address and stimulate more flexible perspectives among students regarding school-related attributes through specific interventions, curricula, informal education, and the daily culture of the school. For example, teachers and other educational agents should (a) promote the importance of learning and new abilities, value effort, and improvement instead of academic marks; (b) reinforce that difficulties and challenges with new knowledge and contents are usual and necessary for improvement; (c) give feedback to all students regarding improvements; (d) propose challenging tasks adapted to different students’ profiles; (e) teach students about basic science, in particular, how the brain can be changed and strengthened through repetition and practice; and (f) introduce information about incremental and entity beliefs with supporting evidence in the field.

This study suggests that working with students to promote incremental and dynamic self-beliefs about their emotional abilities will likely foster their positive future emotional and scholastic experiences, which in turn create conditions for the achievement of better outcomes. This study represents a preliminary effort to investigate the less explored emotion-related ITs, shedding light on how ITEIs relate to students’ emotional and academic outcomes throughout secondary school.

## Data Availability

The data that support the findings of this study are available from the corresponding author upon reasonable request.

## Notes

1. In Portugal, secondary school corresponds to the third level of the International Standard Classification of Education; it lasts for 3 years, comprising the 10^th^, 11^th^, and 12^th^ grades, and covers adolescents ages 15–18 years. Access to secondary school is achieved after the successful accomplishment of 9 years of basic education. After completing secondary school, students can either proceed in their studies, applying to a college educational system (based on academic merit ranking), or enter the labour market.

2. The student’s socioprofessional status was defined according to the parents’ highest level of professional occupation.
